# Knowledge hegemony and epistemic reclamation in Central Asian higher education: a community case study of Eastern scholastic heritage integration, sociological field dynamics, and the conditions for decolonizing economic reasoning across STEM and social science disciplines in Uzbekistan

**DOI:** 10.3389/fsoc.2026.1851253

**Published:** 2026-08-31

**Authors:** Gulinoz Ruziyeva, Temir Olimov, Nargiza Khamroyeva, Salomat Ramazonova, Shokhida Ruziyeva, Maftuna Tosheva

**Affiliations:** 1 Bukhara State University, Bukhara, Uzbekistan; 2 Bukhara State Technical University, Bukhara, Uzbekistan; 3 Asia International University, Bukhara, Uzbekistan; 4 Bukhara State Pedagogical Institute, Bukhara, Uzbekistan

**Keywords:** Al-Farabi, Bourdieu, Central Asia, coloniality of power, cultural capital, curriculum reform, decolonial sociology, disciplinary stratification

## Abstract

This community case study provides a broad sociological account of a pilot curriculum intervention conducted at a regional public university in Uzbekistan, in which modules drawing on Eastern scholastic economic heritage, specifically the thought of Ibn Khaldun, Al-Farabi, Al-Biruni, Al-Ghazali, and Yusuf Khas Hajib were integrated into the general education requirements of both Engineering and Social Science faculties (*N* = 148). Grounded in Bourdieu's field theory and theory of habitus, Fricker's account of epistemic injustice read alongside Spivak's and Dotson's accounts of epistemic violence and silencing, Connell's Southern Theory, the coloniality-of-power framework originated by Quijano and developed by Mignolo, and Wallerstein's world-systems analysis, the study situates the intervention within the layered structures of tsarist colonization, Soviet epistemic modernism, and post-Soviet Westernization that have marginalized Central Asian intellectual heritage in contemporary university curricula. The article makes three interconnected contributions. First, it offers a theoretically grounded account of the sociological mechanisms through which Eastern scholastic economic heritage is excluded from university curricula in post-Soviet Central Asia, arguing that this exclusion constitutes a form of symbolic violence with implications for students' economic mindset formation and professional habitus. Second, it documents the community dynamics of a 4-week heritage integration module, analysing the social processes of habitus disruption, the collapse of the epistemic division of labor between STEM and Social Science fields, and the emergence of epistemic reclamation as a collective social phenomenon. Third, it develops the concept of “field-licensed ignorance” the socially structured and institutionally reproduced disengagement of STEM students from ethical-economic reasoning as a theoretical contribution to the sociology of education. As a single-institution, qualitative case study conducted without a control group and relying substantially on self-report over a 4-week period, the study is exploratory: its findings are analytically rich rather than statistically generalizable, and they support correspondingly modest causal claims. With that caveat, the findings suggest that even brief, well-designed heritage integration interventions can initiate field-positional shifts among both STEM and Social Science students, though sustainable field-level change requires deeper institutional transformation. The study contributes to the literatures on decolonizing economics education, post-Soviet educational transformation, culturally relevant pedagogy in higher education, and the sociology of knowledge, while providing a replicable model for curriculum reform in resource-constrained university contexts across Central Asia and beyond.

## 1 Introduction

The organization of knowledge is never a neutral act. Every university curriculum, syllabus, reading list, and assessment rubric is a political document: a record of decisions about whose knowledge counts, whose intellectual tradition is treated as universal, and whose heritage is rendered local, particular, or pre-scientific (Apple, 2004). Such decisions are shaped by the structures of global academic power that Wallerstein (2004) analyses in world-systems terms, in which institutions of the global North occupy the center as producers of legitimate academic knowledge while those of the global South and semi-periphery are constituted primarily as its consumers and reproducers. Reproduced through citation practices, journal rankings, accreditation frameworks, partnership agreements, and the everyday decisions of curriculum committees, this hierarchy has proved remarkably durable across post-colonial and post-socialist transitions alike.

The global decolonization movement in higher education has generated a substantial literature on these dynamics, from the South African “Rhodes Must Fall” campaign, which began at the University of Cape Town in March 2015 and spread within months to Oxford and beyond (Nyamnjoh, 2016; Chantiluke et al., 2018) on groundwork laid in African decolonial scholarship (Ndlovu-Gatsheni, 2013), to curriculum debates in British universities (Bhambra et al., 2018), Latin American “epistemic disobedience” (Mignolo, 2009), and Indigenous knowledge movements in Aotearoa New Zealand and Canada (Smith, 2021). Connell's (2007) argument that sociological theory is produced overwhelmingly in the global North and presented as universal, while Southern traditions are treated as merely local, has been extended and empirically tested across a widening range of contexts, including through Bhambra's (2014) programme of “connected sociologies” and Smith's (2021) insistence, from a Māori standpoint, that the communities about whom knowledge is produced be recognized as producers of theory and method in their own right. Central Asia, however with its distinctive combination of pre-modern Islamic intellectual heritage, tsarist and Soviet epistemic legacies, and post-Soviet Westernization remains significantly underrepresented in this literature.

This article addresses that gap through a community case study of a curriculum intervention at a regional Uzbek university, analyzed through the lens of the sociology of education. The intervention, which integrated Eastern scholastic economic thinkers into the general education requirements of both Engineering and Social Science faculties, offers a concrete site for examining how knowledge hierarchies are reproduced and, tentatively, disrupted. Precision about location matters here, because decolonization is not a uniform process and the term does not travel unchanged. What decolonial reform demands, and what resistances it meets, differ between the centers of former empires where the debate concerns a canon complicit in empires those states themselves built and the post-Soviet periphery, where universities inherited an epistemic architecture imposed first by a neighboring land empire and subsequently reworked under the sign of a globalizing West (Ndlovu-Gatsheni, 2018; Moosavi, 2020). The analysis developed here is situated in, and speaks primarily to, the second configuration, while drawing comparative lessons for the first.

Central Asia occupies a peculiar position in the global knowledge system, being at once the site of one of the world's most distinguished pre-modern intellectual traditions the Islamic Golden Age, to which it contributed Al-Biruni, Al-Farabi, and Ibn Sina, among others (see Section 4.3) and one of the most thoroughly epistemically colonized regions on earth. That colonization did not begin with the Soviet Union. Tsarist forces took Tashkent in 1865 and Samarkand in 1868, the khanates of Kokand, Bukhara, and Khiva were annexed or reduced to protectorates over the following decade, and the region was administered as Russian Turkestan, a colonial possession in the full sense, until 1917 (Morrison, 2021). Imperial Russia thereby began subordinating the region's own institutions of learning, including the madrasa system through which its scholastic heritage had been transmitted for centuries, well before Soviet power systematized that subordination (Khalid, 2015). Soviet rule then restructured educational institutions, across 70 years, around a Russophone, secularist, and modernist framework that treated pre-modern Central Asian heritage as culturally interesting but scientifically irrelevant (Silova, 2009); universities and academies of science were designed explicitly on Russian metropolitan models, with curricula, personnel, and publication norms imported wholesale from Moscow and Leningrad. Successive alphabet changes Arabic to Latin script in 1928, Latin to Cyrillic in 1940 severed generations of students from direct access to the Arabic, Persian, and Chagatai Turkic corpus in which Central Asian scholarship had been written, copied, and read for roughly a millennium.

Independence in 1991 created the institutional conditions for revising this inheritance, but the revision that occurred was a partial one. Pre-Soviet national narratives were recovered, and historical scholars were celebrated as national cultural icons, yet this recovery was largely performative rather than curricular, enacted in the register of state symbolism figures placed on banknotes from the mid-1990s onward, invoked in official speeches and anniversary commemorations, memorialized in statues and renamed avenues rather than in economics and philosophy syllabi as genuine analytical resources (Adams, 2010). The more substantive shift was, paradoxically, a deepening of Western epistemic influence. From the late 1990s onward, and with renewed intensity after the reform turn of 2017, the Bologna Process (launched in 1999 and increasingly adopted as a reference point for Uzbek reform), World Bank-funded curriculum modernization projects, international accreditation agencies, and the aspiration toward global rankings codified in the national Concept for the Development of Higher Education to 2030 (adopted by presidential decree in 2019) created powerful incentives to align curricula with anglophone academic norms, intensifying the marginalization that tsarist and Soviet rule had begun. These reforms are recognizably neoliberal: they tie institutional legitimacy, funding, and prestige to externally defined metrics rather than to locally defined intellectual purposes (Komatsu and Rappleye, 2017).

Uzbekistan presents a particularly rich case for examining these dynamics. With a population of approximately 37 million and the largest higher education system in Central Asia, enrolling some 42% of the relevant age cohort by 2023, up from 9% in 2016 (World Bank, 2022), the country is expanding university access rapidly at precisely the moment when questions about the content of that education are most urgent. Its 2030 Strategy and 16 national Sustainable Development Goals commit explicitly to an indigenous-informed development path, yet the system continues to reflect, in its basic epistemic architecture, structures inherited from Soviet curriculum design and amplified by post-Soviet Westernization (UN Sustainable Development, 2023; Jafarova et al., 2026). The SDG framework is itself ambiguous here: its content gestures toward locally defined priorities, but its form globally standardized targets, indicators, and reporting instruments belongs to the same family of metrics-driven governance as the neoliberal reforms above, and can plausibly be read as part of that package rather than an alternative to it (Komatsu and Rappleye, 2017).

Recent empirical work underscores the depth of the challenge. Bespalyy et al. (2024), surveying students and faculty across Kazakhstan, Tajikistan, Kyrgyzstan, and Uzbekistan, found that only 56% of Uzbek teachers indicated awareness of the Sustainable Development Goals adopted at the 2015 summit, the lowest figure among the surveyed countries; awareness among students was lower still, and most reported learning about global sustainability challenges through social media rather than formal curricula. Such a disconnect between national policy commitments and classroom reality is symptomatic of a deeper curricular inertia. The host institution for this study is a mid-sized regional public university in Uzbekistan, here anonymized; its heavily technical-vocational orientation, subordinate field position relative to national flagship universities, exposure to strong conformity pressures, and largely regional student intake make it representative of a broad category of Central Asian institutions for which these findings are most directly relevant.

The study pursues four aims. First, it develops a sociologically grounded account of the mechanisms through which Eastern scholastic economic heritage is structurally excluded from university curricula in post-Soviet Central Asia. Second, it documents the community dynamics of a heritage integration intervention, recording how students and instructors respond when dominant epistemic hierarchies are challenged in the classroom. Third, it develops a set of sociological concepts field-licensed ignorance, epistemic reclamation, and habitus disruption with analytical purchase beyond the case examined here. Fourth, it derives practical implications for curriculum reform in Central Asian higher education and contributes to the international literature on decolonizing university curricula.

The article proceeds as follows. Section 2 sets out the context of the study Uzbek higher education, the host institution, its students, and the scholarship on which the intervention drew. Section 3 develops the theoretical framework. Section 4 sets out the methods, covering the research design, the intervention, and data collection and analysis. Section 5 presents the case evidence, Section 6 discusses its implications, and Section 7 offers recommendations for curriculum reform and future research. Section 8 concludes.

## 2 The context of the study: Central Asia and Uzbekistan

Because the argument that follows turns on features of Uzbek higher education that readers outside the region are unlikely to know, this section sets out the context of the study before the framework and the intervention are presented. Under Soviet rule Uzbekistan developed a comprehensive public education system that achieved near-universal literacy and produced substantial numbers of engineers, scientists, and technical specialists. That system was, however, epistemically uniform: it was built around a standardized Russophone curriculum which treated Central Asian cultural and intellectual heritage as historical material to be preserved and celebrated in museums, while scientific and economic education proceeded exclusively within Western and Soviet-Western frameworks. Independence disrupted the linguistic architecture of that system without disturbing its epistemic one. The replacement of Russian with Uzbek as the language of instruction completed for primary and secondary education by the early 2000s and still ongoing in higher education required the rapid development of Uzbek-language textbooks and teaching materials. In many scientific and technical fields these were produced by translating Russian originals rather than by drawing on indigenous scholarly traditions, reproducing in Uzbek the same epistemic architecture that had been established in Russian.

This pattern is not peculiar to Uzbekistan, and a body of work in comparative education helps situate it. Silova and Steiner-Khamsi's (2008) volume on educational reform in the Caucasus and Central Asia documents how international NGOs and donor organizations shaped post-Soviet curriculum reform, often in directions that reinforced rather than challenged Western epistemic dominance, and Komatsu and Rappleye's (2017) analysis of the PISA-based research program shows how globally dominant knowledge regimes reproduce themselves through apparently neutral technical instruments. Osipian's (2009) study of post-Soviet higher education, though concerned with institutional rather than epistemic hierarchies, identifies the structural pressures that push peripheral universities toward conformity with external norms; Kuzhabekova's (2024) review of equity in Central Asian higher education policy notes the persistent tension between national cultural goals and the globally dominant norms of academic production; and Heyneman's (2000) analysis of education and social cohesion identified the erosion of a shared public educational culture as a central risk of the post-socialist transition. Kandiyoti's (2007) account of the “paradoxes of the informal economy” in Uzbekistan, the persistence of traditional social networks beneath the surface of formally modernized institutions applies mutatis mutandis to the educational field, where informal epistemic practices such as the private valorization of indigenous intellectual heritage and the circulation of pre-Soviet texts coexist with formally Western-oriented curricula. The intervention examined here can be read as an attempt to bring those informal practices into the formal curriculum.

The host institution is a mid-sized regional public university, anonymized in accordance with participant consent protocols. Established in the Soviet period around technical and agricultural education, reflecting the planned economy's regional priorities, it added Economics, Law, and Social Sciences faculties after independence while retaining a predominantly technical identity and resource base. At the time of the study it enrolled approximately 8,000 students across 12 faculties, with Engineering and Agriculture remaining the largest and best resourced. In Bourdieu's field-theoretical terms, set out in Section 3, it occupies a subordinate position within the national academic field: it holds less symbolic capital than the flagship metropolitan universities, receives lower funding per student, has a less internationally active faculty, and draws a more locally oriented student body. Field theory predicts that peripheral institutions conform to dominant norms more closely than central ones, since they lack the accumulated symbolic capital that permits successful deviation, and the prediction holds here: the university's curriculum committee was highly attentive to Ministry of Higher Education guidelines and had little appetite for experimentation.

The intervention was therefore doubly unusual—a curriculum innovation in an institution with strong norms against innovation, and an epistemic challenge to a knowledge hierarchy in an institution with strong incentives to reproduce it. That it was approved at all reflects a specific constellation of actors: a newly appointed Vice-Rector for Academic Affairs (the senior officer responsible for curriculum in the Uzbek university structure) with a background in the philosophy of education, and a small working group of faculty spanning Economics, Philosophy, and Engineering who framed the proposal as consistent with national priorities around cultural heritage and identity. The modules were placed in the general education requirement, a block of courses that all students take irrespective of faculty, which made a single intervention reaching both Engineering and Economics cohorts administratively possible. That framing was itself a form of field navigation, securing institutional space by presenting the intervention in terms the dominant field found legible.

The 148 participating students were drawn from two cohorts: 74 second- and third-year Engineering students, spanning Civil, Computer, and Electrical Engineering, and 74 second- and third-year Economics students, spanning Business Economics, Finance, and International Economics. The Engineering cohort was 62% male and 38% female and the Economics cohort 48% male and 52% female, distributions typical of these disciplines in Uzbek universities; over 90% of students in both cohorts were from Uzbekistan, with small numbers from neighboring Tajikistan and Kazakhstan. Structured pre-module interviews with a purposive sub-sample of 24 students, 12 per faculty, revealed marked differences in prior exposure. Economics students had generally encountered the names of Ibn Khaldun and Al-Farabi in mandatory history of philosophy courses—free-standing units disconnected from their economics teaching, a point whose significance is developed in Section 5.1, but fewer than 20% of those interviewed could identify any specific economic concept associated with them. Engineering students had, with very few exceptions, no prior academic exposure to any Eastern scholastic thinker in any context. Several expressed surprise at the suggestion that medieval Islamic scholars had contributed to economic thought; one summarized a widely shared view: “I knew about Ibn Sina as a doctor, but I had no idea he or anyone from that period wrote about economics. The only economics I know is from our textbooks, which are basically Western.”

That asymmetry sits within a wider disciplinary stratification which was, at the host institution, not merely an administrative arrangement but a socially lived reality with clear hierarchical dimensions. Engineering students consistently described their discipline as “serious” and “practical” in contrast to the social sciences, which were “soft” language that directly encodes the field's symbolic hierarchy. Economics students were often dismissive in turn of engineering's perceived “narrowness” and “lack of social awareness.” These are not merely interpersonal attitudes, nor simple in-group favoritism of the kind social identity theory describes (Tajfel and Turner, 1979), but expressed forms of field-level hierarchies that structure resource allocation, career expectations, and curriculum design. The resulting epistemic division of labor the allocation of economic reasoning exclusively to Social Science is both symptom and cause: it deprives Engineering students of the tools for economic reasoning while reinforcing the social science faculties' claim to exclusive ownership of the domain. The social allocation of different forms of knowing to different social positions has a long history in the sociology of knowledge, running from Mannheim's (1936) analysis of the social determination of thought to Young's (1971) “new sociology of education,” its later social-realist restatement (Young, 2008), and Bernstein's (2000) work on the classification and framing of educational knowledge, with Moore and Muller (1999) and Muller and Young (2019) offering tools for understanding why such divisions persist. The particular form examined here, the structural exclusion of STEM students from any ethical-economic thinking, has not to our knowledge been directly analyzed; the concept of field-licensed ignorance developed in this study names it.

This stratification is not a local idiosyncrasy but a regional instantiation of a much older epistemic settlement. The division between “hard,” objective sciences and “soft” social and humanistic knowledge descends from the European Enlightenment's ordering of knowledge and, behind it, from the Cartesian separation of mind from body and of the knowing subject from the world of values an ordering that decolonial scholars identify as a core operation of the coloniality of knowledge, elevating one culturally particular mode of knowing (disembodied, quantified, purportedly placeless) to the status of universal reason against which all other knowledges are measured and found wanting (Quijano, 2007; Mignolo and Walsh, 2018). Soviet scientific modernism reproduced and intensified this settlement rather than challenging it, and it is carried forward today by adherents on both sides of the divide. Even economics positioned locally on the “soft” side of the boundary yet aspiring to the prestige of the “hard” carries that history within itself: as Raworth (2017) shows, the discipline secured its scientific standing in the 20th century precisely by remaking itself in the image of physics, shedding its older ethical and historical dimensions in the process. The disciplinary stratification observed at the host institution is thus the coloniality of knowledge in action, and this matters for what follows: the divide cannot be treated as a neutral administrative fact to be worked around, but must be named as part of what a decolonial curriculum reform contests.

Two bodies of scholarship supplied the resources for a response. The first is the literature on decolonizing economics education, which grew substantially after the 2008 financial crisis exposed the limitations of mainstream models and triggered student-led curriculum reform movements (Colander, 2005; Sen, 1987): the Post-Crash Economics Society, founded at the University of Manchester in 2012, the International Student Initiative for Pluralism in Economics, established in 2014, and related movements in South Africa and Latin America. Academic responses have included proposals for “pluralist” economics education (Dede, 2010) and a growing body of work on the historical exclusion of non-Western economic thought from the canon, of which Raworth's (2017) reconstruction of the discipline around a social foundation and an ecological ceiling is the most widely circulated instance. That such work is now undertaken within the former imperial centers themselves is significant: the University of Glasgow, whose own history and endowments are entangled with empire, hosts a cross-disciplinary Decolonizing the Curriculum community of practice that has documented resources for decolonizing disciplines including economics (Vincent et al., 2024) an institution which continues to celebrate Adam Smith, who taught there, while rarely interrogating the structuring contrast between “civilized” and “savage” nations on which the Wealth of Nations rests and the characterizations of Indigenous American, African, and Asian societies that are, by any contemporary standard, racist (Boer, 2015). The mechanism operating at the center is the one whose peripheral effects this study documents.

The second body of scholarship concerns Eastern scholastic economic thought itself, and it is far more substantial than its curricular presence suggests. Ibn Khaldun's contributions to economic sociology and macroeconomics are well documented (Spengler, 1964; Rima, 2009): his theory of cyclical economic history, his account of the welfare effects of taxation, his labor theory of value, and his analysis of the social foundations of economic productivity are recognized by economic historians as anticipating, and in some respects surpassing, Smith, Ricardo, and Keynes. Hosseini (2003) demonstrates that medieval Muslim contributions to the history of economics have been systematically undervalued by a Western-dominated historiography; Ghazanfar and Islahi (1990) document Al-Ghazali's treatment of price, value, and exchange and its relationship to later scholastic economic thought in medieval Europe; and Chapra (2014) and Greif (2006) establish the depth of the economic thought and practice associated with the Islamic Golden Age, including the institutions of the Silk Road.

How such material is taught matters as much as whether it is included, and here the literature on culturally relevant pedagogy (Ladson-Billings, 1995, 2014; Gay, 2010) provides an empirical foundation: culturally resonant content enhances engagement, knowledge retention, and higher-order thinking, a claim consistent with the learning-sciences finding that new knowledge is built on learners' prior knowledge and cultural resources (Bransford et al., 2000). Paris and Alim's (2017) “culturally sustaining pedagogy” extends the framework to the active role of education in sustaining cultural practices valuable in their own right, independent of their instrumental contribution to academic achievement (Paris, 2012). Because this framework travels from the global North, its use in decolonial contexts requires care: Moosavi (2020) warns that “intellectual decolonization” can itself become a bandwagon tokenistic, Northern-led, and inattentive to Southern scholars and Smith (2021) insists that engagement with marginalized knowledge is decolonizing only when conducted on terms that respect the communities who bear it. What is missing across both literatures is pedagogical: systematic documentation of what happens when Eastern scholastic economic thought is actually integrated into a university curriculum. This study was designed to address that gap, with both of these cautions in view. The framework through which it does so is set out next.

## 3 Theoretical framework

The context set out above poses a set of analytical problems: why a knowledge hierarchy inherited from three successive imperial orders should persist in a curriculum that national policy explicitly commits to indigenizing; why the disciplinary divide between Engineering and Economics is experienced by those on both sides of it as natural rather than imposed; and what would have to change for either to be otherwise. This section assembles the framework used to address those questions, drawing on Bourdieu's field theory, work on epistemic injustice and epistemic violence, Southern and decolonial theory, world-systems analysis, and micro-sociological accounts of classroom interaction.

A note on the positionality of this framework is warranted, since there is an evident irony in analyzing the marginalization of non-Western knowledge with a toolkit assembled largely in the global North: Bourdieu, Fricker, Wallerstein, and Collins are all situated within Northern metropolitan academia. The study responds in three ways. These theorists are placed in dialogue with thinkers from and of the global South Quijano, Ndlovu-Gatsheni, Tuhiwai Smith, Alatas, Santos, and above all with the Eastern scholastics themselves, treated throughout as theoretical resources rather than objects of study (Connell, 2007; Alatas and Sinha, 2017). The colonial matrix of power is credited to its originator, the Peruvian sociologist Aníbal Quijano, rather than only to its best-known anglophone exponent (Quijano, 2000, 2007; Mignolo, 2023). And Walter Mignolo's own position an Argentine scholar writing from and with the South while holding a chair at Duke University exemplifies the navigation between Southern intellectual commitments and Northern institutional locations that Central Asian academics, in their own register, must also perform. Rather than disqualifying the framework, this asymmetry is treated as data about the knowledge system the study analyses.

### 3.1 Bourdieu: field, capital, habitus, and symbolic violence

The sociology of Pierre Bourdieu provides the primary analytical scaffold for this study. Bourdieu's concept of the social field: a structured space of positions defined by the distribution of different forms of capital allows us to understand the university and its disciplinary sub-fields not as neutral educational environments but as arenas of symbolic struggle in which different knowledge traditions compete for legitimacy (Bourdieu, 1984; Bourdieu and Passeron, 1977). In the academic field, as Bourdieu and Wacquant (1992) elaborate, the specific capital at stake is primarily cultural capital in its institutionalized and embodied forms: academic credentials, disciplinary competencies, publication records, citation networks, and, crucially, the habitus of the academically successful agent.

Habitus, in Bourdieu's account, is the durable system of dispositions

“systems of durable, transposable dispositions” (Bourdieu, 1990, p. 53)

that individuals acquire through socialization in particular social positions and that orients their perceptions, judgments, and practices in ways that typically reproduce the social conditions of their formation. For students in Uzbek universities, years of schooling in a curriculum that presents Western economic frameworks as the natural and universal form of economic knowledge produce a habitus of epistemic deference: a disposition to regard Western knowledge as legitimate economic science and indigenous knowledge traditions as culturally interesting but economically irrelevant. This habitus is not the product of conscious prejudice or deliberate exclusion; it is the sedimented outcome of the curriculum's implicit evaluative structure, reproduced across thousands of small interactions between teachers, textbooks, assessment criteria, and students.

Bourdieu and Passeron's (1977, p. 4) concept of symbolic violence names the process by which this arbitrary imposition of one knowledge tradition's dominance is rendered natural and self-evident. Symbolic violence does not require explicit coercion; it operates through the “misrecognition” of cultural arbitrariness as natural necessity (Bourdieu and Passeron, 1977, p. 4). When students in Uzbek universities experience the absence of Ibn Khaldun from their economics curricula not as a political decision but as a natural fact of academic life as simply “how economics is done” symbolic violence is operating at its most effective. The intervention examined in this study is, at one level, an attempt to break this misrecognition: to make visible the arbitrary character of the curriculum's epistemic hierarchy and thereby create conditions for its contestation.

Reay's (2004) extension of habitus theory to educational research is also relevant here. Reay argues that habitus is not a fixed structure but a dynamic system that can be disrupted by encounters with novel social contexts that do not fit the dispositions the agent has acquired. Educational experiences that place students in contact with knowledge traditions, pedagogical styles, or epistemic authorities that diverge from those their habitus has been calibrated to expect can produce disjunctures between habitus and field moments of dissonance that, if sustained and supported, can initiate processes of habitus modification rather than mere disquiet (Reay, 2004, p. 436). The intervention examined in this study is designed to create precisely such moments.

### 3.2 Epistemic injustice, epistemic violence, and silencing

Miranda Fricker's concept of epistemic injustice provides a complementary register operating at a finer grain of social interaction than Bourdieu's field-level analysis (Fricker, 2007). Two of her forms are operative here. Testimonial injustice occurs when prejudice leads a hearer to assign a speaker a deflated level of credibility as a knower because of their social identity or group membership (Fricker, 2007, p. 1); in the curriculum context it operates when Eastern scholastic thinkers are structurally excluded from the position of economic authorities, their contributions treated as interesting but not as scientifically valid on the same terms as those of Western economists. Hermeneutical injustice occurs when a gap in collective interpretive resources puts someone at an unfair disadvantage in making sense of their own social experience (Fricker, 2007, p. 1). Central Asian students who sense often intuitively rather than articulately that their heritage is relevant to the economic questions they study lack the conceptual vocabulary to express or develop that sense, precisely because the curriculum has withheld the interpretive tools that would make it expressible.

Fricker's vocabulary understates what is at stake in one respect. The structural removal of an entire intellectual tradition from a curriculum is more than a maldistribution of credibility; it is closer to what Spivak (1988) names epistemic violence, the violence done to colonized subjects when the frameworks through which knowledge is recognized are built so that those subjects cannot appear within them as knowers at all. Dotson's (2011) development of that line is the more directly useful for this setting. Dotson analyses epistemic violence as a practice of silencing taking two forms testimonial quieting, in which an audience fails to recognize a speaker as a knower, and testimonial smothering, in which speakers truncate their own testimony because they anticipate it will not be taken up and locates its root in pernicious ignorance, a reliable ignorance that causes harm in the situation at hand (Dotson, 2011, p. 238; for an overview applied to higher education settings, see Hosseini, 2025). Both forms are visible in the case evidence: Eastern scholastic thinkers are quieted by a curriculum that does not admit them as economic authorities, and students smother their own intuitions about the relevance of their heritage because the curriculum has taught them that such intuitions carry no academic standing. The study accordingly uses the terms together, with “epistemic injustice” designating the distributive dimension of the phenomenon and “epistemic violence” and “silencing” its structural dimension.

This register also illuminates the asymmetric credibility judgments students apply to Eastern vs. Western scholarly sources. As reported in Section 5, several students questioned whether translations of Eastern thinkers could be trusted as authentic representations of the originals, a source skepticism not applied with anything like the same rigor to Western texts a direct manifestation of testimonial injustice, reflecting an internalized hierarchy of epistemic credibility that operates largely below the threshold of deliberate judgment (cf. Greenwald and Banaji, 1995).

### 3.3 Southern and decolonial positioning

The study writes within a body of Southern and decolonial scholarship, and it is worth stating plainly that this literature supplies the study's stance rather than its analytical instruments. Connell's (2007) Southern Theory argues that the sociological canon Durkheim, Weber, Parsons, Habermas is the product of particular metropolitan centers in Western Europe and North America yet is taught as though universal, while intellectual traditions from the global South are admitted as area studies or as empirical material to be theorized by Northern frameworks rather than as theoretical resources in their own right. Smith (2021) documents how research itself has operated as an instrument of empire, and reclaims research and by extension curriculum as a site of self-determination over whose knowledge counts. Ndlovu-Gatsheni (2013, 2018) analyses how coloniality survives formal decolonization and calls for epistemic freedom as the decolonial project's unfinished business; (de Sousa Santos 2007, 2014, p. 92) names the elimination of alternative ways of knowing epistemicide; and Wallerstein's (2004) world-systems analysis situates the concentration of legitimate knowledge production in core institutions, and the constitution of peripheral ones as reproducers, as a systematic feature of the global capitalist system rather than a local accident.

The genealogy of the framework's central decolonial concept is itself part of that stance. The coloniality of power, together with the colonial matrix of power the interlocking structures of economic, political, gendered, and epistemic domination that constitute coloniality was developed by the Peruvian sociologist Quijano (2000, 2007), who argued that the classification of the world's populations around the idea of race, and the concomitant hierarchization of their knowledges, was constitutive of capitalist modernity rather than incidental to it. Walter Mignolo, the framework's most widely read exponent in the anglophone academy, is explicit that he came to the concept through Quijano (Mignolo, 2023, p. 39–46), and Grosfoguel (2007, 2022) records that Quijano's formulation was itself developed in dialogue with the Black radical tradition, most notably Cedric Robinson's (1983) analysis of racial capitalism. Attending to this genealogy is not a bibliographic nicety: it enacts, within the study's own citation practice, the principle of crediting Southern and marginalized thinkers as originators rather than as footnotes. Of this body of work one concept does analytical duty in what follows Mignolo's epistemic disobedience, the deliberate refusal of the terms of the colonial epistemic contract (Mignolo, 2009, p. 159–160; Mignolo and Walsh, 2018) and it does so as the foil against which this study's concept of epistemic reclamation is defined in Section 6.

### 3.4 Post-Soviet knowledge politics as a specific configuration

The post-Soviet setting introduces a configuration of epistemic dynamics that has been theorized, in different registers, as the coloniality of knowledge (Quijano, 2007), as epistemicide (de Sousa Santos, 2014), and as the denial of epistemic freedom (Ndlovu-Gatsheni, 2018). What distinguishes it is layering: unlike many post-colonial contexts in Africa, South Asia, or Latin America, Central Asian universities confront not a single colonial epistemic legacy but the three successive ones described in Section 2. The shorthand “double marginalization” used throughout this article refers to the twentieth- and twenty-first-century layers, and should be read as resting on the longer imperial foundation beneath them. The analytical consequence is a distinctive form of epistemic uncertainty. Where counterparts in African or South Asian contexts can often invoke a relatively continuous, if internally differentiated and contested, post-colonial intellectual tradition, Central Asian academics and students must navigate between a pre-Soviet heritage celebrated culturally but largely inaccessible scholastically, a Soviet intellectual inheritance discredited politically but still deeply embedded in institutional structures, and a Western-oriented academic culture that offers professional rewards at the price of intellectual autonomy. Silova's (2009) treatment of the “post-socialist” as a specific epistemic condition, rather than simply a transitional moment on the way to Western modernity, is essential to understanding this configuration.

### 3.5 Interaction ritual chains and the sociology of the classroom

Collins's theory of interaction ritual chains (Collins, 2004) supplies the micro-sociological mechanism that field-level analysis leaves unspecified. Collins argues that emotional energy the enthusiasm, commitment, and sense of group solidarity that sustain social practices is generated and regenerated through face-to-face interactions that achieve mutual focus and shared emotional entrainment. Educational settings are, in this view, chains of interaction rituals that either generate or deplete the emotional energy sustaining learning. When students experience their heritage as epistemically legitimate when the traditions they carry are recognized and engaged rather than excluded the energy available for learning is enhanced; when they experience it as illegitimate, that energy is depleted, and with it the motivation for intellectual risk-taking and the development of higher-order thinking.

This mechanism is what makes the module's design consequential rather than merely well-intentioned. The deliberate construction of mixed-faculty groups combining Engineering and Social Science students sought to generate inter-disciplinary emotional energy through repeated face-to-face interaction around shared intellectual objects, the thinkers and their economic arguments (cf. Johansson, 2004, on innovation at disciplinary intersections); the focus group data examined in Section 5 provide evidence that the choice was consequential. Collins thus supplies the interactional mechanism for what the critical pedagogy tradition has long argued on normative grounds: Freire's (1970) critique of “banking” education and his alternative of dialogic, problem-posing enquiry, together with Hooks's (1994) engaged pedagogy, in which the classroom becomes a location where marginalized knowledges and the students who carry them are recognized rather than disciplined, supply the intervention's pedagogical ethics.

### 3.6 The limits of these frameworks and the concepts developed in this study

Each of the frameworks set out above was imported into this study, and none of them, singly or together, proved sufficient to describe what the case evidence showed. Three concepts were developed abductively, in the movement between framework and material described in Section 4.6, first noticed in the pre-intervention baseline, then specified and refined against the module data and because the findings and discussion are stated in their terms, they are named here rather than introduced without warning.

Field-licensed ignorance denotes the socially structured and institutionally reproduced disengagement of a whole cohort from a domain of knowledge, where that disengagement is experienced by its bearers not as exclusion but as natural vocation. It is not captured by symbolic violence, which concerns the misrecognition of a knowledge hierarchy rather than the absence of knowledge; nor by hermeneutical injustice, which names an individual's interpretive disadvantage rather than an institutionally licensed collective condition; nor quite by Dotson's pernicious ignorance, which identifies an ignorance that harms others in testimonial exchange rather than one licensed in its bearers as vocation. Section 6 develops these distinctions.

Habitus disruption denotes the moments of dissonance produced when students encounter epistemic authorities that their habitus has not been calibrated to expect. Bourdieu and Reay supply the mechanism, but not an account of what follows when such disruption is deliberately engineered as curriculum rather than encountered by accident.

Epistemic reclamation denotes the collective social process through which students reconstruct their relationship to their own heritage as a source of legitimate knowledge. It is distinguished from Mignolo's epistemic disobedience, an intellectual-political position adopted by scholars who explicitly refuse the colonial epistemic contract, in that it arises among students holding no prior theoretical commitment, out of experiential encounter rather than principle.

The claim is not that the imported frameworks are dispensable. Bourdieu's field theory structures the analysis throughout, and the epistemic injustice literature supplies the vocabulary in which the baseline condition is described. The claim is that the phenomena this case exhibits fall in the gaps between them.

## 4 Methods

### 4.1 Research design

This study employs a qualitative community case study design (Yin, 2018), in which the unit of analysis is the community formed by the participants in the module students, instructors, and working group members rather than individual actors. The community case study format is particularly appropriate for this research because the phenomena under investigation the social processes of habitus disruption, field-licensed ignorance, and epistemic reclamation are inherently collective: they occur in and through social interaction rather than within individual cognition. The format also aligns with the Frontiers in Sociology Community Case Study article type, which is designed to document and analyze the dynamics of specific communities confronting specific challenges.

The case was selected on the basis of four criteria: (1) the availability of a sustained and well-documented intervention to analyze; (2) the representativeness of the institutional context for a broader category of Central Asian universities; (3) the disciplinary diversity of the participant community, which allowed comparative analysis across STEM and Social Science cohorts; and (4) the willingness of institutional gatekeepers to support rigorous data collection including audio-recorded focus groups and instructor observation logs. The single-case design limits the generalizability of specific findings but enables the depth of analysis necessary to develop and refine the theoretical concepts that constitute the study's primary contribution.

### 4.2 Development of the intervention

The intervention was grounded in three theoretical propositions, each corresponding to a mechanism through which Eastern scholastic heritage can be expected to foster economic mindset development. The first proposition drawing on Ladson-Billings (1995) and Gay (2010) holds that culturally resonant curriculum content enhances student engagement and knowledge retention, and that for Central Asian students, the economic thought of the Islamic Golden Age constitutes a culturally resonant body of material with which standard Western-oriented economics curricula cannot compete. Because this first proposition rests on a literature that originates in the global North, the design deliberately read it alongside decolonial voices that speak from and with the global South: Smith's (2021) insistence that engagement with marginalized knowledge be conducted on terms that respect its bearers; Alatas' analysis of academic dependency and his call for alternative discourses generated outside the Euro-American core (Alatas, 2003, 2006); Andreotti's (2011) actionable post-colonial theory in education; and Ndlovu-Gatsheni's (2018) account of epistemic freedom as the horizon of curricular change. The design also took seriously Moosavi's (2020) genealogy of the “decolonial bandwagon” and his warning that intellectual decolonization can be nominal, tokenistic, and even recolonizing when performed for metropolitan audiences without substantive engagement; the working group treated that warning as a design constraint, reflected in the principles set out in Section 4.4.

The second proposition is drawing on Fricker (2007) holds that providing students with hermeneutical resources that connect their cultural heritage to contemporary economic questions addresses a specific form of epistemic injustice, with consequences for students' sense of themselves as legitimate knowers and economic reasoners. The third proposition is drawing on Quijano (2000), Mignolo (2009), and de Sousa Santos (2014) holds that the encounter with heritage-based economic knowledge can activate what Mignolo calls epistemic disobedience: a willingness to think from and with suppressed knowledge traditions rather than exclusively within the terms set by dominant epistemic frameworks.

The module was developed by an interdisciplinary working group convened by the Vice-Rector for Academic Affairs and comprising five members: a professor of economic history (Economics faculty), a senior lecturer in philosophy of science (Humanities faculty), an associate professor of engineering ethics (Engineering faculty), a doctoral researcher in educational sociology (external collaborator), and a librarian specializing in Central Asian manuscript collections. The working group met weekly for 8 weeks prior to the module's delivery, and its discussions were recorded and subsequently analyzed as data about the institutional dynamics of curriculum reform.

The co-design process was itself a site of productive tension. The economic historian and the philosopher advocated for a historically rigorous approach that would present each thinker within their intellectual and social context before drawing contemporary connections; the engineering ethics professor prioritized contemporary relevance and practical application; the sociologist emphasized the importance of explicit attention to power and epistemic hierarchy. The resolution of these tensions was a structured progression from historical context to contemporary application, with explicit facilitation prompts encouraging students to reflect on why this material was absent from their standard curricula shaped the module's distinctive pedagogical character.

### 4.3 Module structure and content

The 4-week module, titled “Economic Thinking from the Silk Road,” was delivered as a standalone four-credit-hour general education requirement, meeting twice weekly in 90-min sessions. Each week focused on one or two primary thinkers, with a structured progression from historical contextualization to conceptual analysis to contemporary application. Table 1 below summarizes the module structure.

**Table 1:** Module structure, thinker coverage, sociological concepts, and contemporary case exercises.

Wk	Thinker(s)	Core economic concept	Sociological lens (dialogue partner)	Contemporary case
1	Ibn Khaldun (Muqaddimah, c. 1377)	Cyclical economic history; ‘asabiyyah as social capital; labor theory of value; welfare effects of taxation	Social reproduction of economic structures; Bourdieu's social capital	Post-Soviet institutional trust in Uzbekistan's transition economy
2	Al-Farabi (al-Madina al-Fadila) & Yusuf Khas Hajib (Kutadgu Bilig)	Cooperative economic organization; political economy of just governance; reciprocal obligations of rulers and producers	Testimonial injustice in governance discourse; hermeneutical resources (Fricker)	Public procurement ethics and the sociology of bureaucratic authority
3	Al-Biruni (Kitab al-Hind) & Al-Ghazali (Ihya Ulum al-Din)	Empirical market methodology; moral economy; price theory grounded in human need; critique of currency debasement and speculative hoarding	Epistemic disobedience; moral economy (Thompson); embeddedness (Granovetter)	Mobile fintech regulation, currency ethics, and the embedding of digital payment systems in Uzbekistan
4	Silk Road institutions: hawala, waqf, caravanserai, merchant guilds	Pre-modern institutional economics; contract enforcement without formal legal systems; credit and endowment across long-distance networks; information asymmetry solutions	Institutional sociology (Greif); Southern Theory (Connell); epistemic reclamation	Designing a community innovation endowment for a regional SME cluster in Bukhara region

A brief word on the geographic and civilizational provenance of these five thinkers is warranted, because the module's decolonial framing rests on a distinction that is easily blurred and, if left implicit, invites the charge of a merely performative gesture. Three of the five belong, in present-day terms, to Central Asia, and can be reclaimed as Central Asian heritage—and, in Al-Biruni's case, as Uzbek heritage specifically: Al-Biruni was born around 973 in Khwarazm (Kath, near modern Khiva, in present-day Uzbekistan); Al-Farabi was born around 870 in Farab, later known as Otrar, on the Syr Darya in what is now southern Kazakhstan; and Yusuf Khas Hajib was an 11th-century Karakhanid Turkic figure from Balasagun, in modern Kyrgyzstan, who composed the Kutadgu Bilig in Kashgar. The remaining two are thinkers of the wider Islamic Golden Age whose lives unfolded outside Central Asia: Ibn Khaldun (1332-1406) was born in Tunis, in North Africa, into a family of Andalusian Arab descent, and Al-Ghazali (c. 1058-1111) was a Persian scholar born in Tus, in the Khorasan region of what is now northeastern Iran. The module neither effaces this distinction nor annexes the latter two as ethnically or territorially Uzbek. Rather, it situates all five within the connected, trans-regional scholarly ecumene of the medieval Islamic world, in which scholars, texts, and ideas circulated across Bukhara, Baghdad, Nishapur, Cairo, and Córdoba. Two analytically distinct claims are therefore in play, and they are deliberately kept apart: first, that Central Asia produced world-historical thinkers whose economic reasoning its own universities have marginalized, which is a claim of local reclamation; and second, that the broader Islamic Golden Age, of which the region was an integral part, constitutes a shared intellectual heritage on which Central Asian curricula may legitimately draw, which is a claim of civilizational connection. Holding these two claims apart, rather than collapsing geography into civilization, is what distinguishes substantive heritage integration from the tokenistic “decolonial” gesture that (Moosavi 2020) warns against.

Two features of this structure deserve emphasis. First, as the fourth column of Table 1 makes explicit, the Eastern scholastic thinkers were not presented in isolation or as a separate canon: they were placed in structured dialogue with Northern and Western theorists Bourdieu on social capital, Fricker on testimony, Thompson on moral economy, Granovetter on embeddedness, Greif on institutions, Connell on Southern Theory read alongside, and against, the heritage material. This choice was deliberate and reflects a critical-decolonial rather than nativist design logic: the aim was not to remove or erase established thinkers but to bring marginalized thinkers to the table on equal terms, enabling a conversation between traditions in which each can question the other, the approach modeled, in the teaching of sociological theory, by Alatas and Sinha (2017). Second, this dialogic structure meant that the Eastern thinkers appeared as interlocutors within the analytical mainstream of each week's work, rather than as a “heritage corner” appended to it; the significance of this design decision is underlined by the academic literacies evidence discussed in Section 5.1.

### 4.4 Pedagogical approach and design principles

Six design principles guided the module's pedagogical approach, each grounded in the theoretical framework and the specific institutional context.

- **Primary source engagement:** Students were given excerpts from the thinkers' own works in translation (Ibn Khaldun, 1967; Dankoff, 1983), rather than secondary summaries. This choice was deliberate and theoretically motivated: it positioned Eastern scholars as epistemic authorities speaking in their own voice, operationalizing Fricker's insight about testimonial injustice by restoring direct discursive access that the dominant curriculum had withheld.
- **Historical contextualization before contemporary application:** Each session began with a 20-min lecture establishing the thinker's historical, social, and intellectual context, resisting the anachronistic temptation to treat pre-modern thinkers simply as proto-modern economists. This contextualization was followed by conceptual analysis and only then by contemporary application.
- **Mixed-faculty grouping:** Student groups were constituted to include both Engineering and Social Science students, creating conditions for interaction ritual chains (Collins, 2004) across disciplinary boundaries and disrupting the epistemic division of labor by requiring both groups to apply each week's concepts to contemporary case exercises.
- **Explicit attention to epistemic hierarchy:** Facilitation guides included specific prompts encouraging students to reflect on why the material they were encountering had not previously appeared in their curricula. This reflective dimension was designed to activate the denaturalization of field structure: the movement from misrecognition to recognition of the arbitrary character of the curriculum's knowledge hierarchy.
- **Contemporary Uzbek case exercises:** Each week's conceptual work was anchored in a contemporary case exercise drawing on real economic challenges facing Uzbekistan: post-Soviet institutional trust, public procurement ethics, fintech regulation, and regional SME development. This anchoring served to demonstrate the living relevance of Eastern scholastic concepts, not merely their historical interest.
- **Formative and summative assessment:** Weekly structured reflection tasks (assessed against a rubric measuring quality of economic reasoning and evidence of cultural capital revaluation) served both as assessment instruments and as data sources. The final summative assessment was a group presentation applying the semester's conceptual resources to a self-chosen contemporary economic challenge.

### 4.5 Data collection

Four data collection instruments were employed, each chosen to illuminate different aspects of the social processes under investigation.

Structured reflection tasks were completed by all students at the end of each weekly session. Each task asked students to: (a) identify the most significant economic concept they had encountered that week; (b) explain how it related to a contemporary economic issue in Uzbekistan; and (c) reflect on whether and how encountering this material had affected their understanding of what “economic knowledge” is. Responses were analyzed against a rubric developed by the working group, assessing quality of economic reasoning (0–4 scale) and evidence of epistemic repositioning (0–3 scale). Inter-rater reliability was established through double-scoring of a 20% random sample, achieving a Cohen's kappa of 0.74 (substantial agreement).

Collaborative case exercise outputs were collected at the end of each weekly session. Groups of 4–6 students (mixed STEM and Social Science) submitted written analyses of the week's contemporary case exercise. These outputs were reviewed by a faculty panel of three (one from each participating department) and analyzed for evidence of cross-disciplinary reasoning, ethical-economic integration, and innovation in applying historical concepts to contemporary problems.

Focus group interviews were conducted at the end of the module with eight purposively selected groups: four Engineering groups and four Economics groups, each comprising 6–8 students selected to ensure variation in gender, year of study, and prior academic performance. Interviews lasted 60–80 min and were audio-recorded and transcribed verbatim. Interview protocols were developed iteratively, with initial questions focusing on students' experience of the module and later questions probing emergent themes from the reflection task analysis. All transcripts were coded in full and analyzed thematically (Braun and Clarke, 2006); the coding procedure is described in Section 4.6.

Instructor observation logs were maintained throughout the 4 weeks by the two primary instructors, who recorded observations about student engagement, group dynamics, cross-disciplinary interaction, and any notable moments of resistance or breakthrough. These logs were analyzed as a complementary data source that provided instructor perspectives on the same dynamics documented in the student data.

### 4.6 Analysis, ethics, and positionality

Analysis was abductive (Timmermans and Tavory, 2012), moving iteratively between theoretical framework and empirical material: theory was used to identify significant patterns while the analysis remained open to patterns that required theoretical development or revision. The concepts brought to the data habitus disruption, field-licensed ignorance, epistemic reclamation, testimonial injustice functioned as sensitizing concepts rather than fixed categories, and were specified and revised against the material rather than applied to it. Two of them, field-licensed ignorance and epistemic reclamation, did not exist at the outset; they were formulated in the course of analysis to name patterns for which the imported vocabulary proved inadequate, as set out in Section 3.6. The reported findings are accordingly theory-informed rather than purely data-driven, and are presented as such.

Coding proceeded in two passes. The first was an open pass across the full corpus focus group transcripts, structured reflection tasks, collaborative case exercise outputs, and instructor observation logs in which codes were generated from the material without reference to the study's constructs. In the second pass these codes were consolidated and related to the sensitizing concepts, producing the analytic categories reported in Section 5. Member-checking was conducted with four student participants and two instructors, who reviewed summaries of the emerging findings and commented on their accuracy and resonance.

The studies involving human participants were reviewed and approved by the Research Ethics Committee of the host institution, Uzbekistan. The studies were conducted in accordance with the local legislation and institutional requirements, and ethical approval was obtained prior to data collection. All participants provided their written informed consent to participate in this study, including explicit acknowledgment that participation was voluntary and would have no effect on academic assessment. Given the power differential between instructors and students, consent was obtained by a researcher not involved in module delivery. All data are reported in anonymized form, with direct quotations lightly edited for anonymization. The working group member who was also a researcher on this study (the doctoral researcher in educational sociology) maintained reflexive field notes throughout and used these to monitor the potential influence of their dual role on data collection and analysis.

## 5 Case evidence

**Figure 1:**
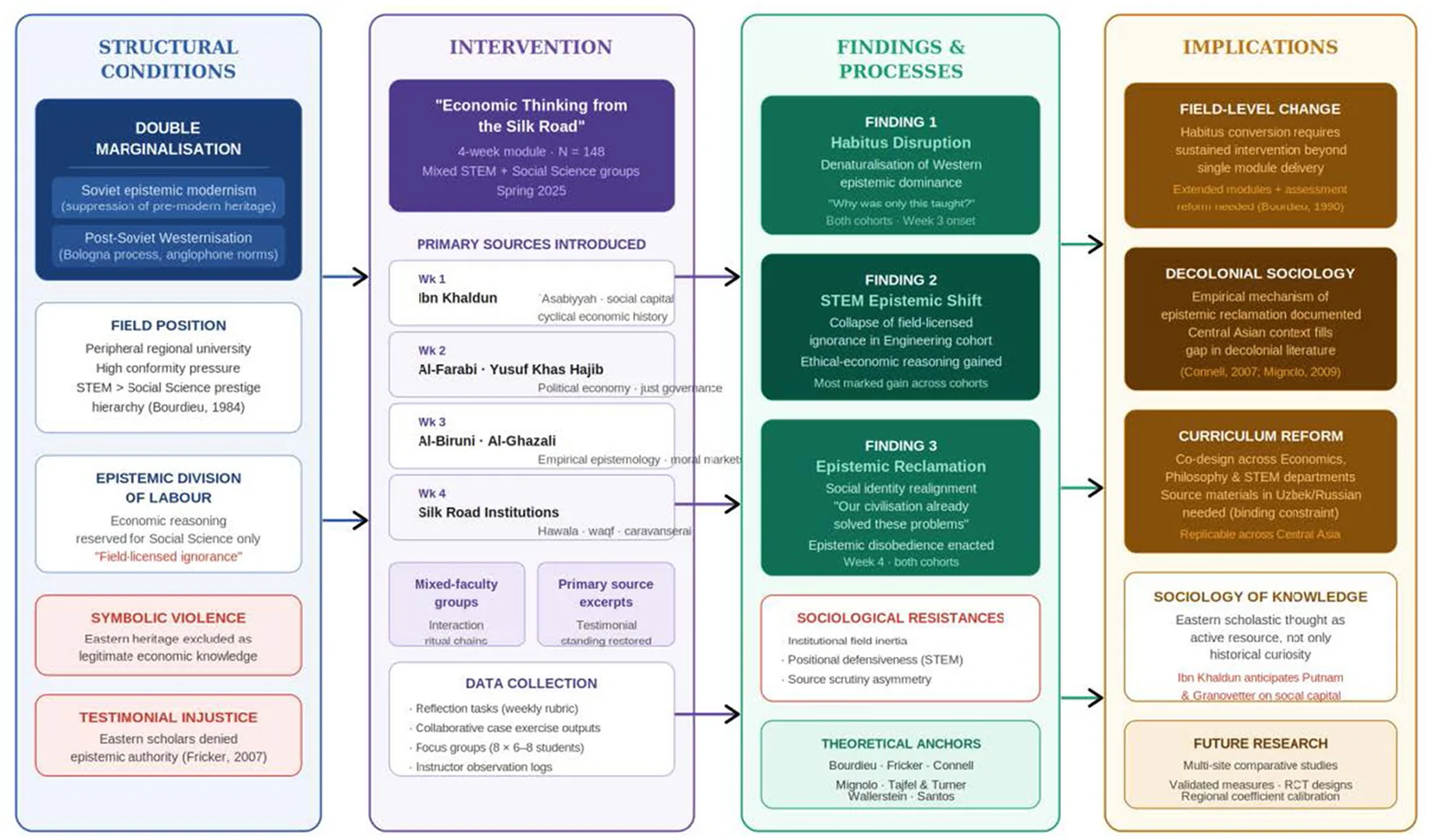
Conceptual framework of epistemic reclamation dynamics in Uzbek higher education: structural conditions **(left)**, intervention design, sociological findings, and implications for curriculum reform and decolonial sociology. Arrows denote analytical flow; red panels indicate sociological resistances. Source: created by the authors.

Figure 1 summarizes the study's conceptual framework, linking the structural conditions set out in Section 2, the intervention design, the findings reported in this section, and their implications for curriculum reform and decolonial sociology.

### 5.1 Pre-intervention baseline

Pre-module structured interviews established a clear baseline against which module effects can be assessed. Engineering students described economic reasoning, in near-universal terms, as lying outside their disciplinary remit not as something they had failed to learn, but as something belonging properly to others. Characteristic responses to the question “Do you feel you have the knowledge and tools to think about economic issues?” included: “That's not my area”; “Economics is for economists”; and “I chose engineering because I'm good at solving technical problems, not because I want to deal with money and markets.” These responses reveal the depth of the epistemic division of labor: it has been so thoroughly internalized that it is experienced not as exclusion but as natural vocation. It is this pattern a socially sanctioned disengagement from economic reasoning that its bearers experience as vocation rather than deficit that the study names field-licensed ignorance.

Economics students displayed a complementary but differently inflected form of epistemic limitation: what we might call “Western epistemic capture” is a genuine competence within the Western canon combined with an inability to conceive of economic knowledge as existing outside that canon. When asked to name an economic thinker who was not Western, the large majority (over 80%) were unable to do so without prompting, despite having completed mandatory history of philosophy courses that included coverage of Al-Farabi. This failure of recall is itself analytically significant, and its likely cause is instructive. The students' prior encounter with Al-Farabi had taken place in a separate, mandatory history of philosophy course: an “add-on” unit with no connection to the way economics was actually taught, practiced, or assessed in their degree programs. Research in academic literacies has long shown that such bolt-on provision fails precisely because it is detached from disciplinary content: learning that is intended to reshape how students think within a discipline must be woven into, and taught alongside, that discipline's own materials and tasks, and cannot function as an extra, optional, or separate element (Lea and Street, 1998; Wingate, 2006). Encountered as a free-standing philosophical curiosity, Al-Farabi could be memorized for an examination and forgotten; encountered nowhere within economics itself, he could not become part of what students understood economics to be. The intervention examined here was designed on the opposite principle heritage material embedded in live economic reasoning and contemporary application and the contrast between the effects of the two kinds of encounter is, as the following findings suggest, consistent with the academic literacies' prediction.

### 5.2 Finding 1: “Now I realize that's a choice someone made”—habitus disruption and the denaturalization of epistemic hierarchy

The most consistent finding across all data sources was a progressive disruption, across the 4 weeks, of students' habitual equation of legitimate economic knowledge with the Western canon. This disruption was most visible in the structured reflection task data, which showed a marked increase in “epistemic repositioning” scores across the 4 weeks for both cohorts: mean scores rose from 0.8 (out of 3) at Week 1 to 2.1 at Week 4 for Engineering students, and from 1.1 to 2.3 for Economics students.

Focus group data illuminate the qualitative character of this quantitative shift. By the module's third week, students in both cohorts were articulating a new form of reflexivity about the curriculum's knowledge hierarchy. A characteristic response from an Economics focus group: “I always assumed that economics started with Smith and Keynes not because anyone told me that, just because those were the only people in our textbooks. Now I realize that's a choice someone made. And I want to know why.” This articulation the movement from implicit assumption to explicit question is precisely what Bourdieu's concept of denaturalization predicts: the disruption of misrecognition, the making-visible of the arbitrary character of the field's knowledge hierarchy.

Instructor observation logs from Week 3 record a notably increased level of spontaneous discussion about curriculum politics, with students in multiple groups raising questions about why they had not encountered these thinkers before, who decides what goes into university curricula, and whether their education had been giving them an incomplete picture of the world. These questions are not merely academically interesting; they represent a shift in the students' relationship to knowledge from passive consumers of an authoritative canon to critical agents who recognize the curriculum as a product of social decisions that can be contested and revised.

### 5.3 Finding 2: “We normally think of this as a purely technical question”—the collapse of field-licensed ignorance among STEM students

The second major finding concerns the specific response of Engineering students to the module. As noted above, Engineering students entered the module with a deeply internalized field-licensed ignorance of ethical-economic reasoning. The trajectory of their reflection task scores across the 4 weeks provides quantitative evidence of a disruption in this disposition: their “quality of economic reasoning” scores rose from a mean of 1.2 at Week 1 to 2.6 at Week 4 (on a 0–4 scale), a larger absolute gain than that observed in the Economics cohort (from 2.1 to 3.1). This finding suggests that Engineering students had more headroom for growth, having started from a lower baseline, but also that the module was effective in activating economic reasoning capacities that field-licensed ignorance had suppressed rather than eliminated.

The collaborative case exercise outputs from Week 3 provide particularly vivid evidence of this shift. The task was applying Al-Ghazali's moral economy framework and Al-Biruni's empirical methodology to the challenge of regulating mobile payment platforms in Uzbekistan elicited responses from Engineering groups that incorporated considerations of social justice, user vulnerability, and the moral obligations of platform operators alongside technical analyses of security architecture and regulatory compliance. One Engineering group's written output included the following observation: “Al-Ghazali's point that prices should reflect genuine human need rather than what people can be made to pay is directly relevant to freemium pricing models in fintech. We normally think of this as a purely technical or business question. But it is also an ethical one, and the ethical question matters for whether the technology actually serves society.” This integration of ethical-economic reasoning into technical analysis is precisely what the concept of field-licensed ignorance predicts will be suppressed by the field's epistemic division of labor and what its disruption makes possible.

Focus group interviews with Engineering students at the module's conclusion revealed that many had found the encounter with Eastern scholastic thought personally significant in ways that extended beyond the module's content. Several students described a revised sense of their own intellectual heritage: “I always thought of myself as an engineer first and an Uzbek second like being Uzbek was just a cultural thing, not related to my professional identity. Now I think there's actually a connection between the way my civilization has thought about problems and the way I approach problems as an engineer. That's a new idea for me.” This revised sense of professional identity the integration of cultural heritage into disciplinary self-understanding represents a form of habitus modification that has implications well beyond economic reasoning.

### 5.4 Finding 3: “My civilization has resources that I can think with”—epistemic reclamation as a collective social process

The most theoretically significant finding of the study concerns the social dynamic that emerged in the Week 4 session on Silk Road institutional innovations the hawala credit system, the waqf endowment, caravanserai network organization, and merchant guild structures. These institutions produced, in both cohorts, a response that was qualitatively distinct from the engagement observed in earlier weeks: a collective emotional resonance that several instructors described in their observation logs as “the room changing.”

The source of this resonance, as focus group interviews subsequently revealed, was the discovery that Central Asian civilization had developed, centuries before the emergence of Western institutional economics, sophisticated solutions to precisely the problems that contemporary institutional economists regard as central: contract enforcement in the absence of formal legal systems, credit provision across long distances without collateral, endowment structures that survive the death of their founders, information asymmetry in long-distance trade. Students discovered, in other words, that their heritage was not merely culturally rich but was institutionally innovative in ways that are directly relevant to the economic challenges of the present. One Economics student articulated this discovery with particular clarity: “I've been studying how to solve problems like contract enforcement and information asymmetry for two years using game theory and institutional economics. And then I find out that merchants on the Silk Road solved these problems nine hundred years ago, and that those merchants were from Uzbekistan. Why was I not taught this? And what else is there in our heritage that I've not been told about?”

This response which we term epistemic reclamation is analytically distinct from the habitus disruption documented in Finding 1. Where habitus disruption involves a questioning of the curriculum's knowledge hierarchy (“why was I only taught this?”), epistemic reclamation involves an active reconstruction of one's relationship to one's heritage as a source of economic knowledge (“my civilization has resources that I can think with”). It represents, in Mignolo's terms, a moment of epistemic disobedience: a refusal to accept the inherited terms of the epistemic contract, enacted not through abstract theoretical commitment but through the concrete experience of discovering that one's heritage is a resource rather than a liability.

Crucially, epistemic reclamation in this study was a collective, not merely individual, process. The mixed-faculty groups created conditions for this discovery to be shared and amplified: Engineering and Social Science students reinforced each other's realizations, with Engineering students' technical frameworks and Social Science students' historical and contextual knowledge combining to produce analyses that neither cohort would have produced alone. Collins's (2004) account of interaction ritual chains helps explain why: the shared emotional energy generated by the discovery of common heritage produced the kind of group solidarity and mutual entrainment that sustains and deepens intellectual engagement.

### 5.5 Sociological resistances and their analytical significance

The intervention also encountered sociological resistances that are analytically important and warrant careful examination. Three distinct forms of resistance were observed, each illuminating a different dimension of the epistemic hierarchy that the module sought to disrupt.

Institutional field inertia manifested in the responses of a minority of STEM faculty outside the working group, who expressed skepticism about the module's academic legitimacy in departmental meetings and informal conversations. The most common objection was that the module was “soft” or “not rigorous” language that encodes the field's symbolic hierarchy, equating rigor with technical-mathematical knowledge and treating humanistic or historical content as epistemically second-rate. This resistance did not prevent the module from being delivered, but it did create an institutional atmosphere that discouraged Engineering students from openly expressing enthusiasm for the module's content in departmental settings outside the module itself. This chilling effect suggests that sustainable field-level change requires not only curriculum innovation but a broader transformation of the institutional discourse about what counts as rigorous academic work.

Positional defensiveness among a minority of Engineering students was visible primarily in the first 2 weeks of the module, before the habitus disruption effects documented in Finding 1 became established. This defensiveness manifested as skepticism about the relevance of the material (“When will I use this as an engineer?”), impatience with historical contextualization (“Why are we spending so much time on the historical background?”), and occasional mockery of the idea that medieval scholars could be relevant to contemporary engineering practice. These responses reflect the internalized symbolic hierarchy of the STEM field, in which the explicit discussion of ethical, social, or historical dimensions of technical problems is experienced as a distraction from “real” engineering. They are, as the resolution of this resistance in most cases by Week 3 suggests, relatively superficial: they represent the defensive expression of a habitus that was more fragile than its confident surface suggested.

Source scrutiny asymmetry was the subtlest but theoretically most interesting form of resistance. As noted in the theoretical framework section, several students in both cohorts, but more frequently in Engineering questioned whether the translated excerpts from Eastern thinkers accurately represented the originals, raising concerns about mistranslation, loss of nuance, and the potential imposition of Western interpretive frameworks on non-Western texts. These concerns are not, in themselves, unreasonable: they reflect genuine issues in the translation and interpretation of pre-modern texts. What is analytically significant is their asymmetry: the same students did not raise equivalent concerns about their Western economics textbooks, which are also translations (from mathematical or English originals into Uzbek pedagogical idiom) and which also involve the imposition of interpretive frameworks that their authors did not share with their current readers. The asymmetric scrutiny applied to Eastern vs. Western sources is a direct manifestation of the testimonial injustice that Fricker's framework identifies: the internalized disposition to regard Eastern knowledge claims as requiring special justification that Western knowledge claims are assumed to have already satisfied.

### 5.6 Instructor perspectives

Instructor observation logs and post-module interviews with the two primary instructors revealed dimensions of the intervention's dynamics that student data alone could not capture. Both instructors reported that the module required them to develop aspects of their own scholarship that their standard teaching had not previously engaged: the economic historian had not previously thought systematically about the pedagogical implications of Ibn Khaldun's work; the engineering ethics instructor had not encountered the Silk Road institutional literature in the context of contemporary engineering ethics. Both described the experience of co-teaching across disciplinary boundaries as intellectually demanding but rewarding in ways that their standard teaching was not.

Particularly notable was the instructors' account of the shift in classroom dynamics across the 4 weeks. In the first week, they reported, the mixed-faculty groups were uncomfortable: Engineering and Social Science students sat physically apart, addressed questions primarily to the instructor rather than to each other, and showed little spontaneous interaction. By Week 3, this pattern had changed substantially: students were routinely consulting across disciplinary lines, with Engineering students asking Economics students to explain market dynamics and Economics students asking Engineering students to analyze the technical dimensions of case scenarios. This shift in the social organization of the classroom is itself evidence of the disruption of the epistemic division of labor: students were no longer acting as if economic reasoning was the exclusive property of one disciplinary field.

## 6 Discussion

The concept of field-licensed ignorance developed abductively from the pre-intervention baseline data and refined through the analytical process represents, we argue, a contribution to the sociology of education's conceptual vocabulary. It designates a specific form of socially structured and institutionally reproduced disengagement from a domain of knowledge in which the disengagement is experienced not as exclusion but as natural vocation. It differs from the familiar concept of “deficit” in that it is not a shortfall relative to some external standard of universal competence but a structured absence produced by the field's symbolic hierarchy, and from ignorance in the ordinary sense because it is the product not of individual failure to learn but of institutional success in reproducing a knowledge boundary.

Field-licensed ignorance is analytically distinct from Bourdieu's concept of symbolic violence in that it focuses on the absence of knowledge rather than its misrecognition, and from Fricker's concept of hermeneutical injustice in that it names a collective and institutionally reproduced phenomenon rather than an individual experience of interpretive inadequacy. It is related to, but narrower than, Dotson's (2011) pernicious ignorance: where Dotson identifies a reliable ignorance that harms speakers in testimonial exchanges, field-licensed ignorance names the institutional licensing of a whole cohort's ignorance of a whole domain, experienced by its bearers as vocation rather than harm. It is closest in spirit to Young's (1971) account of the social organization of knowledge, which identified the school curriculum's knowledge boundaries as products of social power rather than natural facts. It also specifies, at the level of the student, what Bernstein's (2000) analysis describes at the level of curriculum structure: where Bernstein shows how strongly classified boundaries between subjects are institutionally maintained, field-licensed ignorance names what strong classification produces in those who pass through it, not merely unfamiliarity with the neighboring domain but a disposition that experiences the boundary as vocation. Moore and Muller (1999) and Muller and Young (2019) identify the persistence of such boundaries as a problem for curriculum theory without accounting for why those inside them experience them as natural; that is the gap this concept fills.

If field-licensed ignorance names what the intervention disrupted, epistemic reclamation names what emerged in its place. The collective social process through which students reconstruct their relationship to their heritage as a source of legitimate knowledge is analytically distinct from, but related to, epistemic disobedience (Mignolo, 2009) and epistemic justice (Fricker, 2007). Epistemic disobedience, in Mignolo's usage, is primarily an intellectual-political position adopted by scholars who explicitly refuse the terms of the colonial epistemic contract. Epistemic reclamation, as documented here, is a social process occurring in ordinary educational settings among students who have adopted no explicit political position: it emerges from the experiential encounter with heritage as intellectual resource, not from prior theoretical commitment.

The conditions that appear to enable it, on the basis of this case study, include: (1) the presentation of heritage knowledge in primary source form, as direct testimony rather than secondary characterization; (2) the explicit demonstration of the contemporary relevance of heritage concepts to lived economic challenges; (3) the creation of mixed-disciplinary social conditions that allow the emotional energy of discovery to be shared and amplified; and (4) facilitation that encourages explicit reflection on the epistemic hierarchy the module is challenging. The absence of any of these might be expected to impede the process: heritage knowledge presented only in secondary summaries, or without contemporary application, or in socially isolated rather than collaborative settings, is likely to produce intellectual interest rather than the deeper social and identity-level reorientation that characterizes epistemic reclamation.

These conditions connect directly to the framework. The third is an interaction ritual in Collins's (2004) sense: the Week 4 session achieved the mutual focus and shared emotional entrainment his theory specifies, and what instructors recorded as the room changing is what a successful ritual feels like from inside it. The case thus supplies an educational instance of interaction ritual theory and suggests that heritage integration works socially rather than only cognitively the same material encountered privately would, on this account, be unlikely to produce the same effect. The remaining conditions specify what the culturally relevant pedagogy literature leaves general. Ladson-Billings (1995, 2014) and Gay (2010) establish that culturally resonant content improves engagement and retention; this case suggests that resonance alone is insufficient, and that primary-source form, demonstrated contemporary application, and explicit facilitation of reflection on the epistemic hierarchy are what convert resonance into the identity-level reorientation Paris and Alim (2017) call sustaining. It is also worth recording how the design fared against the two cautions set out in Section 2. Moosavi (2020) warns that intellectual decolonization can become tokenistic and Northern-led, and Smith (2021) insists that engagement with marginalized knowledge be conducted on terms the bearing community respects. The co-design process described in Section 4.2 was the response to both, and the reclamation documented here occurred among students who are themselves the heritage community rather than, as Moosavi cautions against, on their behalf.

A further implication concerns the status of Eastern scholastic economic thought within the sociology of knowledge more broadly. The tendency of the international sociology of knowledge to treat pre-modern non-Western intellectual traditions as objects of historical or anthropological interest rather than as theoretical resources for contemporary practice is itself a manifestation of the epistemic hierarchy that Connell's (2007) Southern Theory critiques. This study provides empirical evidence for the contrary claim. Ibn Khaldun's theory of ‘asabiyyah anticipates Putnam's (2000) theory of social capital and Granovetter's (1985) account of economic embeddedness in ways noted by economic historians (Spengler, 1964; Hosseini, 2003) but not yet absorbed into sociology's mainstream conceptual vocabulary. Al-Biruni's empirical methodology anticipates the comparative and historically grounded approach of contemporary institutional economics (Greif, 2006). Al-Ghazali's moral economy framework relates, in a different but analytically kindred register, to Thompson's (1971) account of the moral economy of the English crowd and Scott's (1976) subsistence ethics. And the Silk Road institutional innovations hawala, waqf, the caravanserai represent real-world solutions to problems contemporary institutional economists treat as theoretical challenges: information asymmetry, contract enforcement without formal legal systems, and endowment management across time and distance. Connell's argument is programmatic, asserting that Southern traditions contain genuine theoretical resources; the evidence it requires is the demonstration that specific Southern concepts do analytical work Northern ones do not. The correspondences above are offered in that spirit, as one instance of the programme rather than its completion. The same point bears on the pluralism debate in economics education: Raworth (2017) shows that the discipline's 20th-century narrowing was a choice rather than a discovery, and the material examined here indicates one direction in which that narrowing might be reversed.

For the analysis of knowledge politics in post-Soviet higher education, the findings both support and qualify the existing literature. Silova and Steiner-Khamsi (2008) and Komatsu and Rappleye (2017) document how externally referenced reform agendas reproduce Western epistemic dominance in post-socialist systems, and the curricular inertia described in Section 2 including the low salience of national policy commitments in classroom practice reported by Bespalyy et al. (2024) is consistent with their account. What this case adds is that the resulting configuration is not fixed. The double marginalization of Central Asian intellectual heritage, by Soviet modernism and by post-Soviet Westernization, proved to be a dynamic field configuration that a well-designed intervention could disrupt. The disruption was initiated by a small group of faculty working within existing institutional structures, using existing general education curriculum slots, without additional resources beyond the time invested in module design. The barriers to heritage integration therefore appear less material than symbolic, located primarily in the internalized dispositions of faculty, students, and administrators habituated to treating Western epistemic dominance as natural. They are, in short, barriers of habitus—which also indicates where leverage lies, since Reay's (2004) account of habitus as a dynamic system disruptable by encounters that do not fit it is what the module operationalized.

This qualifies the world-systems reading in one specific respect. Wallerstein's (2004) analysis, applied to higher education, predicts that peripheral institutions will conform to core-defined norms because they lack the symbolic capital that permits deviation, and Section 2 documented exactly that pressure at the host institution, consistent with the conformity dynamics Osipian (2009) and Kuzhabekova (2024) describe. Yet deviation occurred. It required a particular constellation a newly appointed Vice-Rector with relevant intellectual commitments, a working group spanning three faculties, and a framing that presented the intervention in terms the dominant field found legible but that constellation was assembled from ordinary institutional materials. Structural position constrains without determining, and the mechanism of the exception is itself worth theorizing: the intervention drew legitimacy from national heritage policy, which suggests that peripheral institutions may hold resources for deviation that are invisible from the core's frame of reference. The case also bears out Kandiyoti's (2007) account of the distance between formal institutions and informal practice. The private valorization of indigenous intellectual heritage that Section 2 described as circulating informally proved, once given formal curricular space, to be readily activated: what students encountered was not, for most of them, wholly unfamiliar.

The study also illuminates the specific role of the region's layered marginalization in creating conditions for epistemic reclamation. Unlike contexts in which colonial epistemic displacement occurred through a single historical rupture, as in many African post-colonial settings, the Central Asian case involves a more complex sedimentation: the pre-Soviet heritage was already partially suppressed before the Soviet period through the tsarist conquest and colonial administration of the Central Asian khanates (Morrison, 2021), was further suppressed under Soviet rule, and is now doubly overlaid by post-Soviet Westernization. This sedimentation means that the encounter with pre-Soviet heritage can feel as the focus group data suggest like the discovery of something always present but hidden, rather than an encounter with something entirely foreign. That phenomenological character, simultaneously one's own and unfamiliar, is, we suggest, part of what gives heritage integration its distinctive emotional and identity-level resonance. It supports Silova's (2009) contention that the post-socialist names a specific epistemic condition rather than a way-station on the road to Western modernity: these students are not not-yet-modern subjects but subjects negotiating three incompatible epistemic inheritances at once.

Two elements of the framework proved less serviceable than anticipated, and it is worth saying so. de Sousa Santos's (2014) epistemicide, which frames the elimination of alternative ways of knowing as a completed killing, is stronger than what this case exhibits. The heritage in question was suppressed, displaced from the curriculum, and rendered scholastically inaccessible, yet it proved recoverable within 4 weeks by students who had never formally encountered it. What the evidence shows is closer to dormancy than to death, a distinction that matters practically, since it implies the work of recovery is curricular rather than archaeological. Ndlovu-Gatsheni's (2018) epistemic freedom, likewise, names an end-state no general education module can deliver; it functions here as a horizon against which a modest intervention can be located rather than as anything the study can measure. Quijano's (2007) coloniality of knowledge, by contrast, proved applicable at a point the study did not initially anticipate: the STEM–Social Science hierarchy documented at the host institution is, as Section 2 argues, a local instantiation of the Cartesian ordering that decolonial scholarship identifies as coloniality's epistemic operation. The collapse of field-licensed ignorance is therefore a decolonial outcome as well as a pedagogical one, and the two are not separable.

Several limitations must be acknowledged. The single-institution design limits the generalizability of the findings: the field position, faculty composition, student demographics, and administrative culture of the host institution may produce dynamics that do not replicate elsewhere. The relatively small sample (*N* = 148) and the absence of a control group limit the strength of the causal claims the quantitative data can support. Reliance on self-report measures (reflection tasks, focus group interviews) introduces potential social desirability bias, though the anonymous character of the reflection tasks and the focus group format mitigate this concern. The analysis was theory-informed rather than purely data-driven, as Section 4.6 sets out, and the categories under which the findings are reported were shaped by the concepts brought to the material; a differently framed analysis of the same corpus might have foregrounded other patterns. The 4-week duration is short by the standards of most educational interventions, and the follow-up data available at the time of writing are insufficient to assess whether the documented effects were sustained beyond the module's conclusion. The researchers' own positionality also warrants acknowledgment: the working group member who served as a researcher had a clear investment in the intervention's success, which may have shaped data collection and analysis despite the reflexive measures employed. Future research should employ independent evaluators and more controlled designs to strengthen causal inference.

## 7 Implications and recommendations

### 7.1 For curriculum reform in Central Asian higher education

The most direct practical implication of this study is that the integration of Eastern scholastic economic heritage into general education requirements is both feasible and educationally productive within the existing institutional structures of Central Asian universities. The 4-week module format piloted here represents a minimum viable intervention; a six- to eight-week module, with more extensive primary source reading and a larger summative assessment, would allow deeper engagement with the material and is recommended for future iterations. The inclusion of all students are not only those in Social Science programs is essential: the study's most striking findings concern the STEM cohort, and any model that restricts heritage integration to Social Science programs reproduces rather than challenges the epistemic division of labor that the intervention seeks to disrupt.

The co-design process employed in this study involving faculty from Economics, Philosophy, and Engineering, with external expertise in educational sociology and library access to manuscript collections should be treated as a design requirement rather than a luxury. Heritage integration done poorly through superficial gestures to historical thinkers without substantive engagement with their ideas, without attention to the contemporary relevance of those ideas, or through disconnected “add-on” provision of the kind whose failure both this study's baseline data and the academic literacies literature document (Lea and Street, 1998; Wingate, 2006) risks producing the opposite of the epistemic reclamation documented here: a reinforcement of the view that Eastern thinkers are historically interesting but scientifically marginal. The quality of the intellectual engagement matters.

**Recommendation 1:** All universities in Uzbekistan and the broader Central Asian region should integrate a mandatory module on Eastern scholastic economic thought into their general education requirements for all students, regardless of faculty, and should embed this material within, and alongside, core disciplinary teaching rather than delivering it as an optional, extra, or free-standing supplement, since the evidence indicates that bolt-on provision does not transfer (Lea and Street, 1998; Wingate, 2006). A minimum of four credit hours is recommended, with six to eight representing best practice.

**Recommendation 2:** Module design should be led by interdisciplinary working groups that include philosophers, historians, economists, and representatives from STEM faculties, to ensure both intellectual rigor and disciplinary relevance. External collaborators with expertise in educational sociology and Central Asian manuscript traditions should be included where possible.

**Recommendation 3:** Assessment design should include reflection tasks that explicitly evaluate students' capacity to connect Eastern scholastic concepts to contemporary economic challenges, alongside standard measures of content knowledge. This assessment design has the dual function of measuring module effectiveness and reinforcing the module's pedagogical aims.

**Recommendation 4:** National textbook development programs should be initiated to produce high-quality, accessible teaching materials presenting Eastern scholastic economic thought in Uzbek and Russian, at an appropriate level for undergraduate students across disciplines. The existing scholarly literature is rich but largely inaccessible without specialist training; bridging this gap is a practical prerequisite for scale.

### 7.2 For institutional policy and leadership

The study's findings about the role of institutional field inertia in resisting curriculum reform have implications for the leadership strategies required to sustain heritage integration beyond the initial intervention. Senior leaders who wish to support curriculum reform of this kind need to attend not only to formal curriculum structures but to the informal epistemic culture of their institutions: the assumptions about what counts as rigorous academic work, the symbolic hierarchies that position STEM knowledge above humanistic knowledge, and the habituated reluctance of faculty to engage with intellectual traditions outside their primary specialization.

**Recommendation 5:** University administrations should create explicit institutional policies recognizing Eastern scholastic scholarship as a legitimate and valued form of academic expertise, with implications for hiring, promotion, and research funding decisions.

**Recommendation 6:** Faculty development programs should be introduced to build instructors' capacity to teach Eastern scholastic material with confidence and rigor. These programs should be designed to address not only content knowledge but also the epistemic anxieties that arise when academics are asked to teach outside their primary disciplinary training.

**Recommendation 7:** University libraries should be resourced to acquire and make accessible primary and secondary source materials on Eastern scholastic economic thought, including digital access to manuscript collections held in Uzbek, Tajik, and Iranian repositories.

### 7.3 For the international higher education community

The study's findings have implications that extend beyond Central Asia to the international higher education community and to the institutions accreditation agencies, ranking systems, international partnership bodies that shape the epistemic environment within which Central Asian universities operate.

**Recommendation 8:** International accreditation frameworks should be revised to recognize and value the integration of non-Western intellectual traditions into university curricula, rather than treating such integration as marginal or supplementary. Accreditation processes that privilege citation metrics and publication norms anchored in anglophone knowledge systems inadvertently reinforce epistemic Westernization.

**Recommendation 9:** International university partnerships between Central Asian institutions and Western universities should include explicit provisions for joint curriculum development that draws on both partners' intellectual traditions, rather than treating the partnership as a unidirectional transfer of Western knowledge to Central Asian institutions. Decolonizing initiatives now under way within former imperial centers themselves (Vincent et al., 2024) offer natural counterparts for such genuinely bidirectional partnerships.

**Recommendation 10:** Research funding bodies should prioritize comparative studies of heritage integration interventions across Central Asian institutions, using controlled research designs and validated measures of economic reasoning and epistemic repositioning, to build the empirical evidence base for policy recommendations.

### 7.4 For future research

This study raises a number of questions that future research should address. The most urgent is the question of whether the effects documented here particularly the epistemic reclamation process are sustained over time, or whether they fade as students return to curricular environments that continue to reproduce Western epistemic dominance. Longitudinal follow-up studies tracking participants' epistemic attitudes, career choices, and professional practices over 2 to 3 years following the module would provide crucial evidence on this question. Multi-site comparative studies, including institutions with different field positions (flagship metropolitan universities, private universities, technical institutes), different geographic and cultural contexts within Central Asia, and different intervention designs, would provide the generalizability that the present single-case design cannot achieve.

The concept of field-licensed ignorance also merits further theoretical development and empirical testing. Does it operate in the same way in other disciplinary contexts for example, among arts and humanities students who are field-licensed to ignore scientific and technical reasoning? Does its intensity vary with the strength of the field's symbolic hierarchy? Is it more or less susceptible to disruption in different institutional contexts? These questions have implications not only for the specific challenge of heritage integration but for the broader sociology of knowledge and education.

## 8 Conclusion

This article has developed a broad sociological account of a community case study examining the integration of Eastern scholastic economic heritage into the general education requirements of a regional Uzbek university. Drawing on Bourdieu's field theory and account of symbolic violence, Fricker's epistemic injustice framework read alongside Spivak's and Dotson's accounts of epistemic violence and silencing, Connell's Southern Theory, the coloniality-of-power framework originated by Quijano and developed by Mignolo, Wallerstein's world-systems analysis, and Collins's interaction ritual chains, it has analyzed the structural conditions of layered marginalization—tsarist, Soviet, and post-Soviet that have rendered Eastern scholastic thought invisible in contemporary Uzbek curricula, the social dynamics of an intervention that sought to disrupt those conditions, and the theoretical implications for the sociology of education, decolonial sociology, and culturally relevant pedagogy.

Three theoretical contributions have emerged. First, the concept of field-licensed ignorance of the socially sanctioned disengagement of STEM students from ethical-economic reasoning, produced by the field's symbolic hierarchy and experienced not as exclusion but as natural vocation provides a more precise tool for understanding the epistemic division of labor in higher education than existing concepts have offered. Second, epistemic reclamation, the collective process through which students reconstruct their relationship to their heritage as a source of legitimate economic knowledge identifies and names a phenomenon likely to be more widespread than the current literature recognizes, and specifies the conditions under which it occurs. Third, the analysis of layered marginalization the suppression of Eastern scholastic heritage by tsarist colonial administration, then by Soviet modernism, and then by post-Soviet Westernization provides a framework for understanding the Central Asian context that goes beyond both post-colonial analysis, which cannot account for the Soviet layer, and transition theory, which cannot account for the pre-Soviet ones.

On the practical plane, the study demonstrates that heritage integration is feasible and educationally productive within existing institutional structures, requiring no additional resources beyond the investment in module design. The 10 recommendations offer concrete guidance for curriculum designers, administrators, accreditation bodies, and international partners.

The scholars of the Islamic Golden Age produced economic insights of genuine intellectual power and enduring relevance. The social processes through which those insights have been rendered invisible in the universities of the civilization that produced them are what this study has documented. The sociology of education has a distinctive and urgent role in the work of reclamation, not as a discipline standing outside the process, but as one of its active intellectual participants.

## Data Availability

The original contributions presented in the study are included in the article/supplementary material, further inquiries can be directed to the corresponding author.
